# Case Series: Large B-cell Lymphoma With IRF4 Rearrangement Involving the Lung and Tonsil in Two Different Patients

**DOI:** 10.7759/cureus.76758

**Published:** 2025-01-01

**Authors:** Ahmed Abdulrahim, Ahmed Sabri, Bryan Teruya, Changzhao Li, David Cantu

**Affiliations:** 1 Pathology, Creighton University School of Medicine, Omaha, USA; 2 Pathology, University of Nebraska Medical Center, Omaha, USA; 3 Hematopathology, Hematogenix Laboratory Services, Tinley Park, USA; 4 Dermatopathology, Naples Pathology Associates, Naples, USA; 5 Pathology, CHI Health Creighton University Medical Center, Omaha, USA

**Keywords:** irf4 rearrangement, large b cell lymphoma, lymphoma, non-hodgkin lymphoma, tonsilitis

## Abstract

Large B-cell lymphoma (LBCL) with interferon regulatory factor 4 (IRF4) rearrangement is characterized by the monotonous proliferation of medium- to large-sized cells with strong expression of IRF4/multiple myeloma oncogene 1 (MUM1). It predominantly affects children and young adults with a median age of 12. The reported disease sites include head and neck lymph nodes, the Waldeyer's ring, and the gastrointestinal tract. Here, we report two cases of LBCL with IRF4 rearrangement in 73-year-old and 71-year-old patients. The first patient presented with a lung nodule, while the second patient presented with right palatine tonsillitis. To our knowledge, LBCL with IRF4 rearrangement involving the lung in an elderly patient has not been previously reported.

## Introduction

Interferon regulatory factor 4 (IRF4)/multiple myeloma oncogene 1 (MUM1) is a transcription factor that is known to regulate late-stage B-cell differentiation by downregulating BCL6 expression [1,2]. Rearrangements of IRF4 have been identified in low-grade B-cell lymphomas, plasma cell neoplasms, and T-cell lymphoproliferative disorders [3-7]. IRF4 rearrangement in diffuse large B-cell lymphomas (DLBCL) is uncommon. The first case was reported in 2001, showing IGH translocation involving 6p25 (IRF4) in a screening of 173 patients with B-cell lymphomas [8]. In 2011, Salaverria et al. elaborated in great detail the cytogenetics, gene expression profile, tumor biology, and clinical characteristics of large B-cell lymphoma with IRF4 rearrangement (LBCL-IRF4) as a new entity, affecting mainly children and young adults who were previously diagnosed either as DLBCL, GCB type, or follicular lymphoma (FL), grade 3 [9]. Consistently, in 2013, Liu et al. described a group of pediatric FL cases involving Waldeyer’s ring and lymph nodes carrying frequent IRF4 breaks or IGH/IRF4 fusion and uniformly expressing MUM1 [3]. The median age of presentation was 12 years, with a reported age range between 4 and 79 years. Due to its distinct clinical, pathological, and biological profiles, the WHO described LBCL-IRF4 as a new entity in the 2017 revised edition [10]. The majority of the LBCL-IRF4 cases are found in lymph nodes of the head and neck region, the Waldeyer's ring, and, less commonly, the gastrointestinal (GI) tract [3-9]. Involvement of atypical sites has been reported in two cases, including the inguinal region and the cerebrospinal fluid [11,12]. Herein, we report an unusual case of LBCL-IRF4 in a 73-year-old female, with the lung being the primary site of the disease, and another case of LBCL-IRF4 in a 71-year-old female, with the right palatine tonsil being the primary site of the disease.

## Case presentation

Case 1

The patient is a 73-year-old female with a past medical history of GERD, COPD, CHF, asthma, and tobacco dependence who presented for follow-up of pulmonary nodules that were identified on a prior CT scan due to chronic obstructive pulmonary disease (COPD) exacerbation. Family history is significant for lung cancer in her mother, pancreatic cancer in her sister, stomach and bladder cancers in her grandparents, and leukemia in her son. The patient is currently an active smoker. A review of symptoms revealed fatigue, chronic cough, and dyspnea on exertion. A complete blood cell count showed normocytic anemia (hemoglobin 11.1 g/dL). A 1.7 cm irregular nodule in the superior aspect of the right lower lobe was identified and noted to have increased in size since the prior study performed three months ago. No mediastinal or hilar lymphadenopathy was noted. A subsequent PET CT scan showed significantly increased metabolic activity within the previously identified pulmonary nodule involving the medial upper right lower lobe along with hypermetabolic lymph nodes within the mediastinum, which were highly suspicious for a malignant process. The patient underwent endobronchial ultrasound bronchoscopy (EBUS) of lymph nodes from stations 11L, 7, 4R, and 11R, which were negative for carcinoma. Bronchial alveolar lavage (BAL) was also negative for carcinoma. To further investigate the disease, a right lower lobe lung CT-guided percutaneous needle biopsy of the lesion was performed.

**Figure 1:**
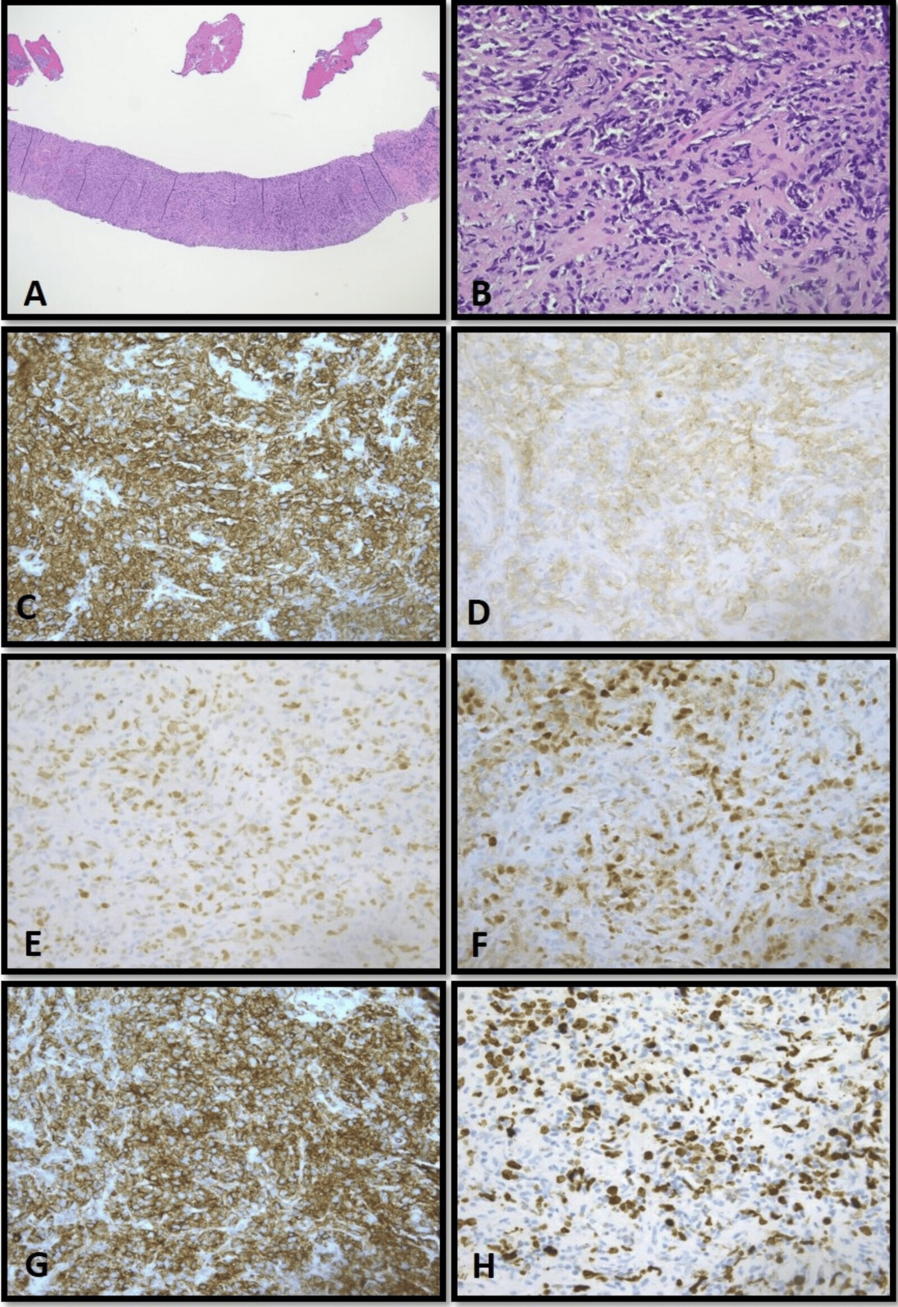
Case 1: Representative pictures of H&E staining and immunohistochemical staining of the lung tissue biopsy sections. (A) H&E staining, 40×; (B) H&E staining, 400×; (C-H) immunohistochemical staining of CD20, CD10, BCL6, MUM1, LCA, and KI-67 sequentially, 400×. H&E: hematoxylin and eosin.

Hematoxylin and eosin (H&E)-stained sections of lung nodule biopsy (Figure 1) showed lymphoid tissue with proliferation of medium- to large-sized atypical cells in a diffuse pattern. The tumor cells have open chromatin with small, basophilic nucleoli. Initial immunostains showed that tumor cells were positive for CD45RB, CD20, CD23 (positive in a subset of tumor cells but no FDC meshwork seen), CD10, BCL6, CD30 (positive in a subset of tumor cells), and MUM1, but negative for CK AE1/AE3, CK7, CK20, TTF1, CD68, chromogranin, synaptophysin, p40, CD3, CD5, BCL1, and BCL2, indicating a preliminary diagnosis of DLBCL, GCB type. The Ki-67 proliferation index was virtually 40%. However, since tumor cells show co-expression of CD10, BCL6, and MUM1, it raised the possibility of large B-cell lymphoma with IRF4 rearrangement (LBCL-IRF4). The paraffin-embedded tissue was sent for molecular cytogenetics testing.

A fluorescent in situ hybridization (FISH) study was performed utilizing the LSI IGH/MYC t(8;14) dual fusion translocation probe with CEP8, the LSI IGH/BCL2 t(14;18) dual fusion translocation probe, the LSI BCL6 (3q27) major and alternate breakpoint dual color break-apart probe, the LSI MYC(8q24) dual-color break-apart probe, and a custom IRF4/DUSP22 (6p25.3) dual color break-apart probe with CEP6. The custom IRF4/DUSP22 (6p25.3) dual color break-apart probe detected an abnormality of both copies of the IRF4/DUSP22 (6p25.3) locus in 41.5% of the interphase cells analyzed. Specifically, studies exhibited a rearrangement of one copy of the IRF4/DUSP22 locus with deletion of the 5’ (telomeric) probe of the other IRF4/DUSP22 locus. Additional studies were negative for IGH/MYC t(8;14)(q24;q32), IGH/BCL2 t(14;18)(q32;q21), and BCL6 (3q27) and MYC(8q24) rearrangements. The diagnosis of large B-cell lymphoma with IRF4 rearrangement was then made. A bone marrow biopsy showed normocellular bone marrow with trilineage hematopoiesis. There was no morphologic, immunohistochemical, or flow cytometric evidence of bone marrow involvement by LBCL.

The patient was treated with R-CHOP (rituximab, cyclophosphamide, doxorubicin, vincristine, and prednisone) chemotherapy. A PET-CT five months later showed complete metabolic resolution of the pulmonary nodule and associated hypermetabolic mediastinal lymphadenopathy. Another CT chest follow-up after three months was also negative for lung nodules.

Case 2

The patient is a 71-year-old female with a past medical history significant for an endometrial carcinoma s/p hysterectomy, diabetes mellitus, and tremors. She presented with a gradually worsening sore throat with painful swallowing and cough. The patient did not have a fever or chills; however, she noticed white patches on her right tonsil. She also had mild hoarseness of voice. No history of smoking, drug, or alcohol abuse. Family history is not contributory. A physical exam revealed nasal congestion and postnasal drip. The right tonsil was enlarged and inflamed with exudate. The neck was supple with some tenderness in the right adenopathy. Courses of amoxicillin, amoxicillin-clavulanic acid, and clotrimazole were tried with no improvement. Covid-19 test, throat culture, and mononucleosis testing were all negative.

A neck CT scan revealed enlargement of the right palatine tonsil and a region of isoattenuation and hypertrophy of tissue density in the right pharyngeal mucosal lymphoid tissue. A PET-CT scan showed focal asymmetric marked hypermetabolism in the right palatine tonsillar region, suspicious for the neoplastic process. The patient underwent flexible laryngoscopy, and a right tonsil biopsy was obtained.

**Figure 2:**
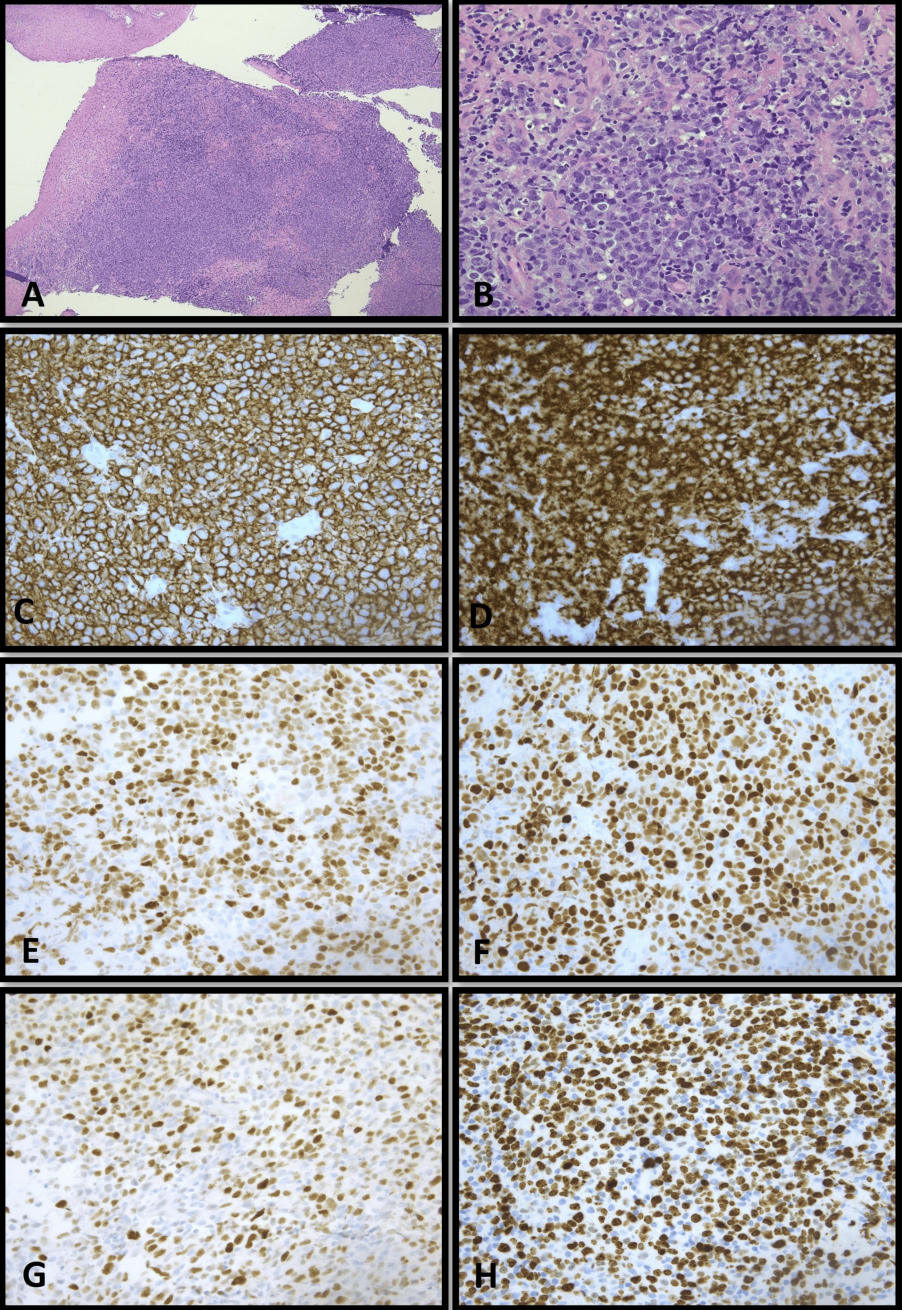
Case 2: Representative pictures of H&E staining and immunohistochemical staining of the tonsillar tissue biopsy sections. (A) H&E staining, 40×; (B) H&E staining, 400×; (C-H) immunohistochemical staining of CD20, CD10, BCL6, MUM1, c-MYC, and KI-67 sequentially, 400×. H&E: hematoxylin and eosin.

H&E-stained sections of the right palatine tonsil (Figure 2) showed effacement of the normal architecture, replaced by sheets of large lymphoid cells with eosinophilic bubbly cytoplasm and large, irregular nuclei with multiple small nucleoli. Immunohistochemical stains showed tumor cells positive for CD20, CD10, BCL-6, BCL-2, MUM-1, C-MYC, and with a high Ki-67 index (80%). The tumor cells were negative for CD3, CD30, CD5, and BCL-1. CD21 demonstrated follicular dendritic cell meshwork throughout. PAS, GMS, and Gram stains highlighted normal oral flora.

Molecular cytogenetic studies detected a rearrangement of the IRF4/DUSP22 (6p253) locus in 92% of the interphase cells analyzed. The overall findings are consistent with LBCL-IRF4.

The patient had received three cycles of R-CHOP and showed a complete response to chemotherapy. She underwent a consolidative/adjuvant external beam radiation involving the right tonsil. Her last PET/CT exam showed complete resolution of the previously hypermetabolic palatine tonsil.

## Discussion

IRF4 rearrangement is seen in approximately 0.05% of DLBCLs [10]. However, its prevalence is much higher (21%) in pediatric patients (<18 years of age) with FL or DLBCL [13]. It most commonly presents with limited disease stages (stage I and stage II), including isolated lymph nodes or tonsillar enlargement. The current defining clinicopathological features for LBCL-IRF4 are the young age of the patient, the location of the disease, and the characteristic immunohistochemical and cytogenetic profile. Tumor cells are commonly positive for CD10 (66% of cases), BCL2 (63% of cases), BCL6 (94% of cases), and MUM1 (100% of cases) [9]. A minority of cases are also positive for CD5 (30%) [9]. This presentation in elderly patients is not typical and may be under-recognized.

In the first case, primary mediastinal LBCL is an important differential diagnosis since the patient had hypermetabolic mediastinal lymph nodes on PET scan. The morphological features and immunophenotype of primary mediastinal LBCL can have significant overlap with LBCL-IRF4. The tumor cells can express MUM1, BCL6, and CD10 (less frequently) [10]. In addition, a weak CD30 expression can be present in >80% of cases. Approximately 70% of cases of primary mediastinal LBCL also express CD23. Therefore, it is very difficult to differentiate between primary mediastinal LBCL and LBCL-IRF4 based on immunophenotype in this case. A thorough literature search did not find any cases that report primary mediastinal LBCL carrying IRF4 rearrangement. On the other hand, there are no cases reporting CD30 expression in LBCL-IRF4. However, based on the cytogenetic finding, we think the LBCL-IRF4 is the most appropriate diagnosis. 

A subset of high-grade FL can have a CD10-negative but MUM1-positive phenotype. These tumors usually lack BCL2 translocation [14]. They need to be distinguished from LBCL-IRF4 with a follicular pattern. Since LBCL-IRF4 can also be negative for CD10, the rearrangement of IRF4 is reliable evidence to support a diagnosis of LBCL-IRF4. Although coexpression of germinal center markers (CD10 and/or BCL6) and MUM1 is not uncommon in DLBCL, NOS, it is usually a useful clue for screening IRF4 rearrangement to have proper subclassification of the disease. Cytogenetically cryptic IRF4 translocations involving IGH (the most common), IGK, and IGL loci have been reported [9]. Both DLBCL, NOS, and LBCL-IRF4 can have BCL6 rearrangement, but MYC and BCL2 rearrangement are virtually absent in the latter [9].

It is also important to differentiate LBCL-IRF4 from pediatric FL if the tumor presents with a follicular growth pattern in children or young adults [15,3]. Pediatric FL often has a similar clinicopathological presentation, including age at diagnosis, limited stage of disease, site of disease, lack of BCL2 translocation, and immune profile. However, it lacks rearrangement or overexpression of IRF4/MUM1 [3,9]. In addition, pediatric-type follicular lymphoma is morphologically characterized by serpiginous follicles with abundant starry-sky histiocytic, which are absent in LBCL-IRF4 [10].

LBCL-IRF4 is believed to be a neoplasm of germinal center B cells. However, it shows distinct molecular biology when compared to other LBCLs. A prior study showed that a set of 27 genes differentially expressed in LBCL-IRF4 demonstrated very high prediction accuracy (96.5%) in cross-validation, indicating its distinct gene expression signature and underlying molecular mechanism [8]. Next-generation sequencing in young patients (<26 years old) revealed that the most frequently mutated genes in LBCL-IRF4 are IRF4 (76%), CARD11 (35%), and CCND3 (24%). This differs from that reported in DLBCL-NOS: SOCS1 (27%), KMT2D (23%), and BTG1, EZH2, GNA13, MYD88, and PIM1 (14%) [16].

The NF-kB signaling pathway is likely to be involved in the molecular pathogenesis of LBCL-IRF4, as some of the mutations are known to drive the constitutive activation of NF-kB [16]. This is consistent with the finding that a significant portion of differentially expressed genes (29%) in LBCL-IRF4 are NF-kB downstream target genes [16]. In addition, somatic mutations of BCL6 affecting the IRF4 binding site in LBCL-IRF4 may explain the loss of suppression of BCL6 expression by IRF4 under otherwise physiological conditions [9,16]. Frequent BCL6 breaks are also observed in 35% of cases [9,16]. The molecular cytogenetic studies in our case also detected an enhancement of the 5’ (telomeric) BCL6 gene region at 3q27, which contains the BCL6 alternate breakpoint region (ABR) in the interphase cells. Specifically, the enhanced 5’-BCL6 signals were larger in size and brighter in intensity than the typical 5’-BCL6 signals but were not clusters of punctate signals that could be quantified. The significance of this finding is currently unknown. LBCL-IRF4 also demonstrates a distinct copy number alteration profile, characterized by frequent 17p/TP53 deletions (25%) without TP53 gene mutations, as well as gains of chromosome 7 (45%) and 11q12.3-q25 (35%) [16].

LBCL-IRF4 is generally indolent and responds well to chemotherapy or radiation [9,13,16,17]. The five-year survival and five-year event-free survival rates were reported to be 100% and 93%, respectively, following current treatment protocols for mature B-cell non-Hodgkin lymphomas [9,16]. In children, a post-excision watch-and-wait strategy has been successful in cases with isolated disease [3]. Prognostic data on LBCL-IRF4 in older patients are lacking due to the limited number of reported cases. A recently published case of LBCL-IRF4 indicates CDKN2A/2B deletion might be associated with poor response to R-CHOP therapy [18]. Other factors that may be associated with worse outcomes include age >18 years, high LDH levels, TP53 mutations, high genetic complexity, and gain 1q21.1-q44 [16].

## Conclusions

In this article, we reported two cases of LBCL-IRF4. This diagnosis in the elderly is not typical and may be under-recognized. One case is presented with a lung nodule, which is also an unusual presentation of this diagnosis.
